# Light Intensity-dependent Variation in Defect Contributions to Charge Transport and Recombination in a Planar MAPbI_3_ Perovskite Solar Cell

**DOI:** 10.1038/s41598-019-56338-6

**Published:** 2019-12-27

**Authors:** Shinyoung Ryu, Duc Cuong Nguyen, Na Young Ha, Hui Joon Park, Y. H. Ahn, Ji-Yong Park, Soonil Lee

**Affiliations:** 1 Department of Energy Systems Research, Ajou University, Suwon, 16499 Korea; 2 Faculty of Engineering Physics and Nanotechnology, VNU University of Engineering and Technology, Vietnam National University, Hanoi, Vietnam; 3 Department of Physics, Ajou University, Suwon, 16499 Korea; 4 Department of Electrical and Computer Engineering, Ajou University, Suwon, 16499 Korea

**Keywords:** Solar cells, Applied physics, Surfaces, interfaces and thin films

## Abstract

We investigated operation of a planar MAPbI_3_ solar cell with respect to intensity variation ranging from 0.01 to 1 sun. Measured *J*-*V* curves consisted of space-charge-limited currents (SCLC) in a drift-dominant range and diode-like currents in a diffusion-dominant range. The variation of power-law exponent of SCLC showed that charge trapping by defects diminished as intensity increased, and that drift currents became eventually almost ohmic. Diode-like currents were analysed using a modified Shockley-equation model, the validity of which was confirmed by comparing measured and estimated open-circuit voltages. Intensity dependence of ideality factor led us to the conclusion that there were two other types of defects that contributed mostly as recombination centers. At low intensities, monomolecular recombination occurred due to one of these defects in addition to bimolecular recombination to result in the ideality factor of ~1.7. However, at high intensities, another type of defect not only took over monomolecular recombination, but also dominated bimolecular recombination to result in the ideality factor of ~2.0. These ideality-factor values were consistent with those representing the intensity dependence of loss-current ratio estimated by using a constant internal-quantum-efficiency approximation. The presence of multiple types of defects was corroborated by findings from equivalent-circuit analysis of impedance spectra.

## Introduction

In recent years, a perovskite solar cells (PSCs) emerged as an intriguing new addition to the list of novel solar cells (SCs) that either challenge or complement already mature silicon SCs^1–3^. Although PSCs can have power conversion efficiency (PCE) as high as 25.2%^4^, they have inherent issues of large hysteresis, poor reproducibility, and limited stability. Previous studies report various degrees of success in improving hysteresis, reproducibility, and stability^5–15^, however, these issues remain to be fully resolved. From a materials point of view, these issues arise from diverse morphological and compositional variations of halide perovskite materials^6,16–19^, crystallization kinetics of halide perovskite grains^5,16,18–21^, various defects and/or remnant of precursors^16–20^, and internal redistribution of ionic species within halide perovskite layers^6,16–19^. Additionally, inorganic materials that are used in conjunction with halide perovskite layers for charge transport and extraction can result in extra interface defects^5,22,23^. Some of these material issues are closely correlated to one another. For example, grain size and/or grain boundary can affect the types and amounts of defects^16,18–20^. Many previous studies, therefore, focused on enlarging grain size^5,16,18,20^ and passivating grain boundary^5,16,18^. Similarly, interface engineering was applied to either passivate interface defects or establish proper energy barriers at the interface^5,22,23^. Finally, judicious selection of precursors and anionic species was found to be efficient in alleviating hysteresis problems due to ionic species^5,6,9^.

Defects in PSCs can affect charge transport and recombination of photo-generated carriers. For example, there were reports that *J*-*V* characteristics of hole-only methylammonium lead iodide (MAPbI_3_) devices were consistent with traits of trap-assisted space-charge-limited current (SCLC)^24–26^. Trap-assisted carrier recombination, which is often called Shockley-Read-Hall (SRH) recombination, is a common mechanism that dominates loss of photo-generated carriers and eventually limits PCEs of various types of SCs^22,27^. Because SRH is a first-order process, it can be distinguished from the second-order recombination process by examining light-intensity dependence of SC operation^22,28^. Studies have evaluated various types of novel SCs in this regard, however, there is a gap in our knowledge of PSC operation. Thorough studies on light-intensity dependence of short-circuit currents^21,29–31^, open-circuit voltages^21,22,31–35^, and ideality factors of PSCs have been scarce^22,36^.

Previously, we attributed variations in Electrochemical Impedance Spectroscopy (EIS) responses and current density-voltage (*J*-*V*) characteristics among PSCs with different types of hole-extraction layers (HEL) to discrepancies among interface- and bulk-defect distributions^37^. Moreover, we tried preliminary quantification of interface- and bulk-defect distributions by using the device simulator SCAPS. In this work, we investigated the light-intensity dependence of IS responses and *J*-*V* characteristics of a planar PSC to understand the role defects play in the operation of these devices. Specifically, PSC that consisted of a MAPbI_3_ active layer, a Cu-doped NiO_x_ HEL, and a PCBM electron-extraction layer was examined. This device structure was used to take advantage of an ohmic contact between MAPbI_3_ and PCBM layers^34,38^.

Interestingly, all the *J*-*V* curves corresponding to diverse illumination levels ranging from 0.01 to 1 sun showed qualitatively identical composition of SCLC and modified Shockley-equation sections. To our knowledge, there are no previous reports of Mott–Gurney’s power law SCLC from working PSCs. The modified Shockley-equation model is a convenient representation of the operation of various types of SCs^22,32,35,36,39^, but rarely analyzed further to identify recombination processes in working PSCs. In this study we evaluated intensity-dependent evolution of recombination processes from in-depth analysis of modified Shockley-equation parameters. Moreover, we obtained corroborating evidence for such recombination-processes from the intensity dependence of loss-current ratio that was estimated under the assumption of a constant internal quantum efficiency (IQE). The constant-IQE assumption allowed us to not only discuss recombination-order variation in a consistent manner, but also estimate IQE at each light intensity.

The PCEs of the PSC we studied in this work did not change substantially with respect to illumination light intensity. Specifically, when light intensity was varied from 1 to 0.01 sun of AM 1.5 G light, PCE remained in the range of 10.5–12.2%. This finding that PCE is sustained under weak illumination expands the potential application of PSC such that it can be used under indoor lighting. Moreover, PSC operation will be less susceptible to limits of lower light intensity conditions such as on cloudy days or in shaded areas.

## Results and Discussion

**Figure 1:**
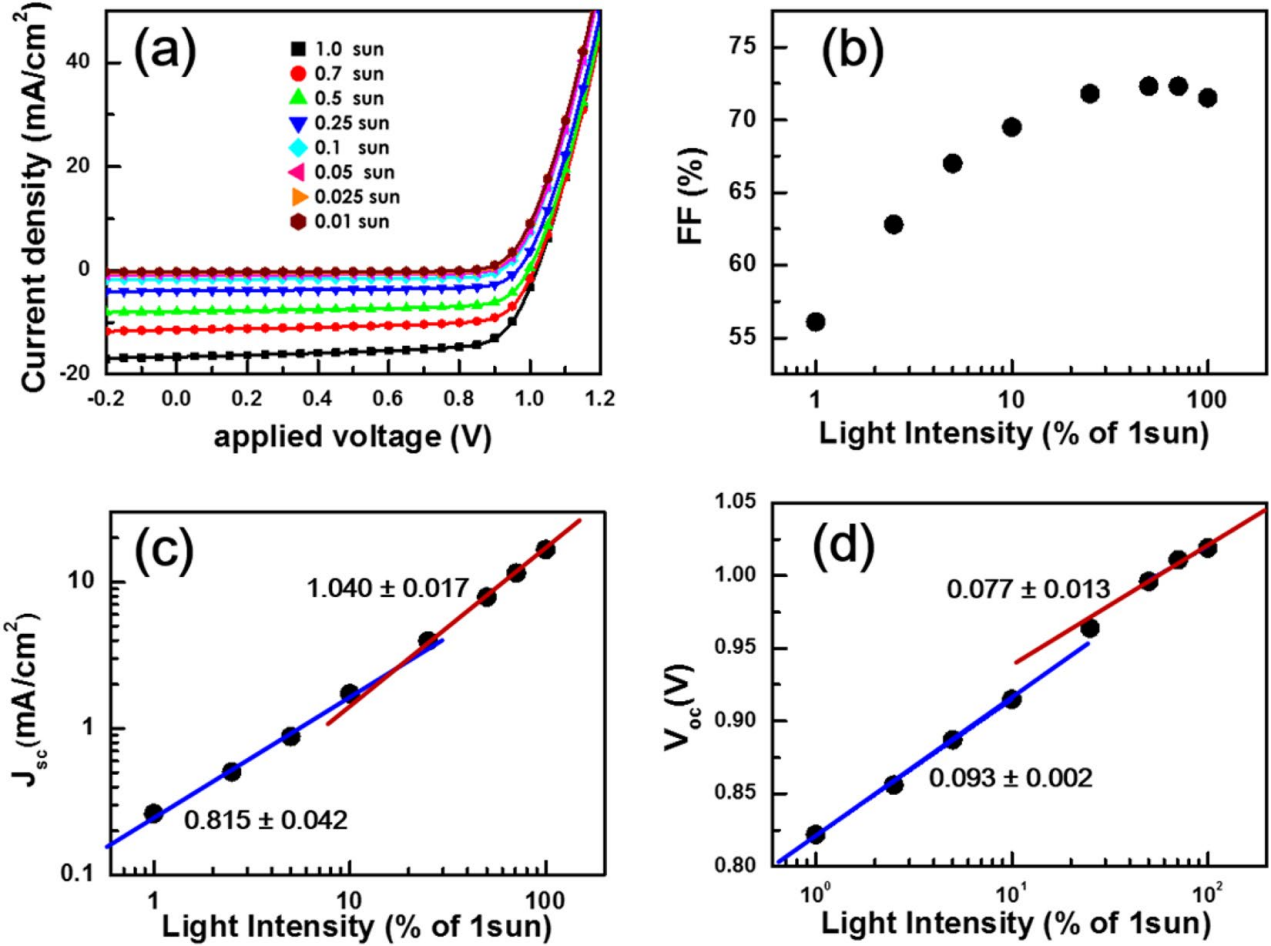
Light intensity dependence of *J*-*V* characteristics of the PSC (**a**) and corresponding solar cell parameters: fill factor FF (**b**), short-circuit current density *J*_sc_ (**c**), and open-circuit voltage *V*_oc_ (**d**). FF that monotonically increased up to the intensity of 10 mW cm^−2^ became saturated at higher (≥25 mW cm^−2^) intensities. Both the variations of *J*_sc_ and *V*_oc_ with respect to illumination intensity in a log-log scale showed two linear segments that correspond to low- (≤10 mW cm^−2^) and high-intensity (≥25 mW cm^−2^) illumination, respectively. All operation parameters of the PSC are listed in Table S1.

Figure 1(a) shows systematic variation in *J*-*V* characteristics of the PSC with respect to the illumination intensity of a solar simulator, which was adjusted using a set of neutral filters. Both short-circuit current density *J*_sc_ and open-circuit voltage *V*_oc_ grew larger with increase in light intensity from 0.01 to 1 sun. Close examination of the *J*-*V* curves showed an intriguing distinction between SC-parameter variations under low- (≤10 mW cm^−2^) and high-intensity (≥25 mW cm^−2^) illumination as shown in Fig. 1(b–d). Fill factor (FF), for example, initially showed a monotonic increase under low illumination, but a saturation behavior under high illumination. In addition, slope of the linear *J*_sc_ variation with respect to light intensity in a log-log scale was 0.815 and 1.040 under low- and high-intensity illumination, respectively. Similarly, variation of *V*_oc_ with respect to light intensity in a semi-log plot showed two linear segments with respective slopes of 0.093 and 0.077 corresponding to low- and high-illumination. Such observations indicated that different mechanisms were at work on the operation of the PSC under low- and high-intensity illumination^21,22,29–35^.

**Figure 2:**
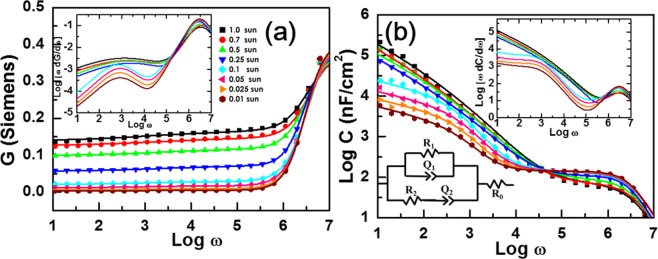
Illumination-intensity dependence of EIS spectra of the Perovskite solar cell. Symbols are respectively semi-log and log-log plots of measured conductance (**a**) and capacitance (**b**) with respect to angular frequency. Lines are the best-fit curves according to the inset equivalent-circuit model. Upper insets corresponding to the derivatives of EIS spectra showed clear distinction between low- and high-illumination operations; moreover, indicated contributions of two processes with respective time constants that differed by three orders of magnitude.

Another evidence for dependence of PSC operation on illumination-intensity was provided from EIS. Figure 2 shows semi-log plots of conductance *G* and log-log plots of capacitance *C* with respect to angular frequency ω. Both *G* and *C* spectra that we were able to fit to an equivalent circuit model in the lower inset showed subtle light-intensity dependence. For example, *G* spectra that hardly changed up to 0.1 sun started to show conspicuous increase under high-intensity operations. In the case of *C* spectra, the most noticeable distinction between low- and high-illumination operations occurred in the slope of low-frequency parts. Specifically, log C-log ω plots increased almost linearly in response to frequency decrease under high illumination, however, showed quasi-saturation behaviors under low illumination. Distinction between illumination dependence under low- and high-intensities were more prominent in the spectra of derivatives of *G* and *C* as shown in the upper insets of Fig. 2. Typically, each peak in such spectra corresponds to a specific time constant that is commonly represented by a combination of capacitive and resistive elements in equivalent circuit models^40–47^. Accordingly, appearance of two peaks in the derivative spectra of *G* and *C* indicate that both slow and fast processes with the respective time constants of ~6 ms and ~2 μs were at work under low illumination. On the contrary, contribution of the slow process and its light-intensity dependence became less prominent under high illumination. One noticeable feature of our equivalent-circuit model is the presence of a parasitic resistance connected in series to the two pair combination of a constant phase element (CPE) and a resistance^40,41^. We noted that a shunt resistance, the other common type of parasitic resistance, was not necessary to fit measured EIS spectra.

**Figure 3:**
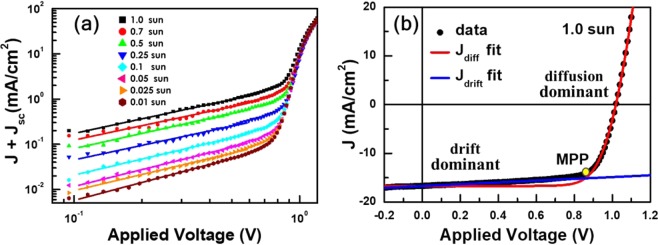
(**a**) Illumination-intensity dependence of diode current *J* + *J*_sc_ with respect to applied bias voltage in a log-log plot. Small diode currents in low-bias range showed slightly super linear power-law dependence and, became larger in response to higher light intensity. On the contrary, diode currents showed conventional exponential dependence on applied voltage in high-bias range above the maximum power points regardless of illumination intensities. (**b**) Measured *J* were fitted to diffusion- and drift-current models in low- and high-bias ranges, respectively. Demarcation between diffusion- and drift-dominant currents occurred in the vicinity of the maximum power point *P*_max_.

It is common to fit *J*-*V* curves of SCs to the Shockley diode equation modified with parasitic resistances for quantitative comparison^32,36,39^. However, we were unable to fit the *J*-*V* curves in Fig. 1 by using such a model. In particular, fitting of the *J*-*V* curves for applied voltages above the maximum power points resulted in large discrepancies in low-bias ranges. The equivalent circuit that we used to model the *G* and *C* spectra in Fig. 2 suggested that shunt resistance effects were insignificant in the operation of the PSC and, therefore, could not be the source of low-bias discrepancies. In order to visually augment the peculiar low-bias characteristics, we transformed the *J*-*V* curves into the log-log plots of *J* + *J*_sc_ versus applied voltages in Fig. 3(a). In high-bias range above maximum power points, *J* + *J*_sc_ showed conventional diode-like exponential dependence on applied voltages regardless of illumination intensities. On the other hand, small *J* + *J*_sc_ in low-bias range, which grew larger in response to increased light-intensity, showed distinctly different power-law dependence on bias voltages. Exponents of power-law dependence were slightly larger than 1 at all illumination intensities, but roughly inversely proportional to light intensity.

Generally, exponential *J*-*V* characteristics of SCs are consistent with depletion approximation that is applicable when width of the active layers are much thicker than that of the depletion zone^36^. In such cases, current is dominated by diffusion of charge carriers and can be represented by the characteristic exponential functional form: *J*_diff_ (*V*) = *J*_diff,0_[exp(*qV*/*k*_*B*_*T*) − 1]. Because increase in forward bias results in narrower depletion zone^36,48^, the depletion approximation becomes more appropriate to account for exponential *J*-*V* characteristics in a high-bias range. On the contrary, power-law dependence in low-bias range is a distinct signature of dominant drift current contributions. In the simplest case, internal electric fields that are linearly proportional to applied voltages drive currents so that ohmic drift currents can appear. However, the aforementioned power-law dependence with exponents larger than 1 indicate that a space-charge-limited currents (SCLC) mechanism was at work to result in non-ohmic drift currents^36,49^.

Another important mechanism that contributed to *J*-*V* characteristics of the PSC was carrier recombination. Because of defects in the perovskite device^16–20^, recombination currents consisted of contributions from both bi- and mono-molecular processes with different exponential dependence on applied bias voltages: ${J}_{{\rm{rec}}}(V)={J}_{{\rm{rec}},0}^{{\rm{bi}}}[\exp (qV/{k}_{B}T)-1]+{J}_{{\rm{rec}},0}^{{\rm{mono}}}[\exp (qV/2{k}_{B}T)-1]$. The diffusion and bimolecular-recombination currents can be merged into a single term to result in conventional diode *J*-*V* characteristics. Moreover, the addition of monomolecular-recombination currents to a conventional diode equation can be approximated by introducing an ideality factor *n*, the values of which lies between 1 and 2: ${J}_{{\rm{diff}}}(V)+{J}_{{\rm{rec}}}(V)\approx {J}_{0}[\exp (qV/n{k}_{B}T)-1]$ (see Fig. S1 in the Supplementary Information)^36^. We note that such an approximation is valid, in particular, in high bias ranges. Conversely, recombination order *β* can be deduced from apparent ideality factor *n* corresponding to high-bias *J*-*V* characteristics: *β* = 2/*n*^36,50^.

Consequently, we represented *J*-*V* characteristics of the PSC with parasitic series resistance *R*_s_ as follows:

1 $$J(V)={J}_{{\rm{d}}{\rm{r}}{\rm{i}}{\rm{f}}{\rm{f}}}^{{\rm{S}}{\rm{C}}{\rm{L}}{\rm{C}}}(V-J\cdot {R}_{{\rm{s}}})+{J}_{0}[\exp \{\frac{q(V-J\cdot {R}_{{\rm{s}}})}{n{k}_{B}T}\}-1]-{J}_{{\rm{s}}{\rm{c}}}.$$

Validity of Eq. (1) was confirmed by fitting the low- and high-bias *J*-*V* characteristics to respective approximations corresponding to dominance of SCLC and exponential components: *J*_low_(*V*) ≈ *B*(*V* − *J*_low_*R*_s_)^*η*^ − *J*_sc_ and *J*_high_(*V*) ≈ *J*_0_[exp{*q*(*V* − *J*_high_*R*_s_)/*nk*_*B*_*T*} − 1] − *J*_sc_ where *B* and *J*_0_ are constants. Figure 3(b) shows results of applying this fitting for the case of 1-sun illumination. Demarcation between diffusion- and drift-dominant approximations occurred in the vicinity of the maximum power point (MPP). Similarly, at all other light intensities, good fitting results were obtained for *J*-*V* curves by using SCLC- and diffusion-dominant approximations in low- and high-bias ranges demarked by MPPs, respectively (see Figure S2 in the Supplementary Information).

**Figure 4:**
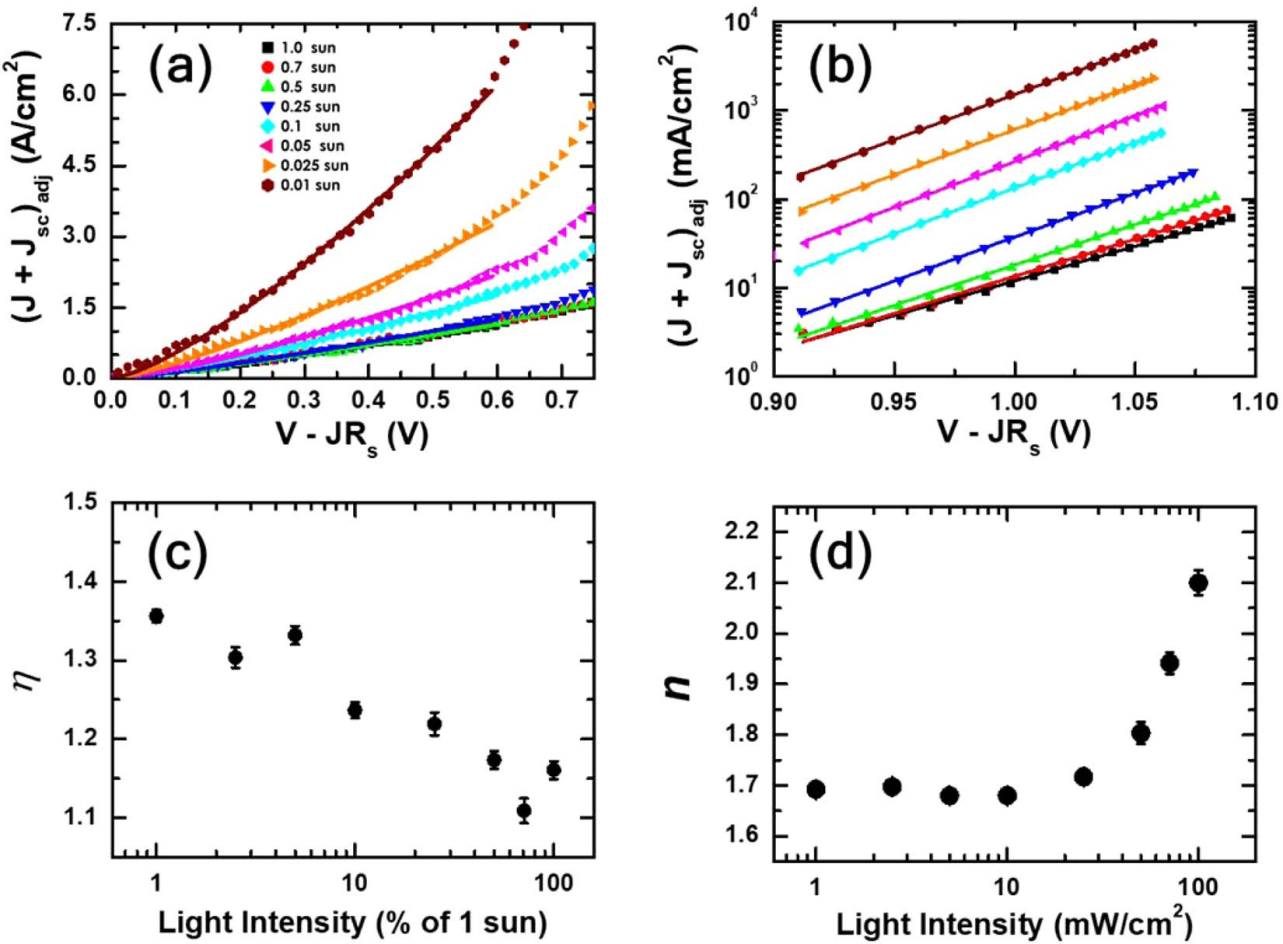
Adjusted currents *J* + *J*_sc_ versus internal voltages *V − J·R*_s_ together with corresponding best-fit curves (solid lines). We used adjusted currents to separate intensity-dependent evolutions of charge-carrier transport and recombination contributions from apparent changes of currents in direct proportion to light intensity. Currents in low bias ranges dominated by drift process were fitted to SCLC approximation (**a**). In high bias ranges, currents dominated by contributions of diffusion and recombination processes were fitted to modified Shockley-equation approximation (**b**). Variation of exponent *η* of the power-law SCLC showed that low-bias drift currents became quasi-ohmic at high illumination intensities (**c**). On the other hand, variation of ideality factor *n* of the modified Shockley equation indicated that recombination currents in high-bias ranges became dominated by monomolecular contributions near 1-sun illumination (**d**).

To illustrate intensity dependent contributions of drift and recombination currents to PSC operation, we transformed the *J*-*V* characteristics into new plots of adjusted *J* + *J*_sc_ versus internal bias voltages *V* − *J·R*_s_ in Fig. 4(a,b). Adjusted currents were defined as measured currents divided by light intensity normalized to 1-sun illumination intensity *L*_1−sun_: (*J* + *J*_sc_)_adj_=(*J* + *J*_sc_)/(*L*/*L*_1−sun_) for *J* + *J*_sc_ measured under the illumination intensity of *L*. The use of adjusted currents allowed us to separate intensity-dependent evolution of drift and recombination mechanisms from changes of apparent currents in direct proportion to illumination intensity. For example, adjusted (*J* + *J*_sc_) versus (*V* − *J·R*_s_) characteristics in low-bias ranges were fitted to the power-law drift current model, and values of exponent *η* that characterizes SCLC at each light intensity were determined. Similarly, values of an ideality factor *n* that characterizes recombination-process order at each light intensity were determined by fitting adjusted (*J* + *J*_sc_) versus (*V* − *J·R*_s_) characteristics in high-bias ranges to the modified Shockley-equation model.

Figure 4(c,d) show changes in values of *η* and *n*, corresponding to solid lines in Fig. 4(a,b), at different light intensities on semi-log plots. We note that *η* decreased in response to increase in light-intensity. Specifically, *η* that was 1.36 at intensity of 0.01 sun became as small as 1.16 at 0.8-sun illumination. This reduction of *η* indicate that light illumination induced changes in defect-related internal charge distribution and suppressed SCLC components in low-bias currents^36,49^. Arguably, sufficiently large illumination intensity resulted in quasi-ohmic drift currents. In the case of *n*, two distinct variations appeared at low- (≤0.1 sun) and high-intensity (≥0.25 sun) illumination. Initially *n* remained in the range of 1.62 – 1.65 up to the illumination intensity of 0.1 sun, however, started to increase monotonically at intensities larger than 0.25 sun. We argue that *n* of ~1.6 was indicative of mixed contributions of mono- and bi-molecular recombination to high-bias currents that were approximated by a modified Shockley equation^36,48,50^. Additionally, we attributed monotonic increase of *n* to growing contributions of monomolecular recombination currents. In particular, *n* of ~2 was hallmark for the dominant contribution of monomolecular recombination to currents in high-bias ranges.

For alternative elucidation of recombination-loss variation with respect to light intensity, we estimated short-circuit and recombination currents with an approximation of constant *IQE*, in which we assumed *IQE*(*λ*) under *f*-sun illumination was an intensity-dependent constant *ζ*_f-sun_ in the wavelength range from *λ*_min_ to *λ*_max_, but zero elsewhere. With this approximation we were able to express short-circuit currents as^51,52^

2 $${J}_{{\rm{s}}{\rm{c}},{\rm{f}}-{\rm{s}}{\rm{u}}{\rm{n}}}=f\cdot {\zeta }_{{\rm{f}}-{\rm{s}}{\rm{u}}{\rm{n}}}\cdot {\int }_{\lambda \,min}^{\lambda \,max}\frac{\lambda }{1240}{\Phi }_{1-{\rm{s}}{\rm{u}}{\rm{n}}}(\lambda )LHE(\lambda )d\lambda ,$$

where *λ* was given in nm, *LHE*(*λ*) was a light harvesting efficiency, Φ_*sol*,1−sun_(*λ*) was light intensity at 1 sun, and *f*·Φ_*sol*,1−sun_(*λ*) was that at *f* sun (see [1] in Supplementary Information). We note that maximum short-circuit currents ${J}_{{\rm{s}}{\rm{c}},{\rm{f}}-{\rm{s}}{\rm{u}}{\rm{n}}}^{max}$ would have been obtained if *ζ*_f-sun_ was 1. In other words, loss currents resulting from recombination *J*_loss,f-sun_ were defined as the difference between ${J}_{{\rm{s}}{\rm{c}},{\rm{f}}-{\rm{s}}{\rm{u}}{\rm{n}}}^{max}$ and *J*_sc,f-sun_. Accordingly, we estimated recombination-loss ratio ${J}_{{\rm{l}}{\rm{o}}{\rm{s}}{\rm{s}},{\rm{f}}-{\rm{s}}{\rm{u}}{\rm{n}}}/{J}_{{\rm{s}}{\rm{c}},{\rm{f}}-{\rm{s}}{\rm{u}}{\rm{n}}}^{max}$ from *J*_sc,f-sun_ and ${J}_{{\rm{s}}{\rm{c}},{\rm{f}}-{\rm{s}}{\rm{u}}{\rm{n}}}^{max}$: ${J}_{{\rm{l}}{\rm{o}}{\rm{s}}{\rm{s}},{\rm{f}}-{\rm{s}}{\rm{u}}{\rm{n}}}/{J}_{{\rm{s}}{\rm{c}},{\rm{f}}-{\rm{s}}{\rm{u}}{\rm{n}}}^{max}=1-{J}_{{\rm{s}}{\rm{c}},{\rm{f}}-{\rm{s}}{\rm{u}}{\rm{n}}}/{J}_{{\rm{s}}{\rm{c}},{\rm{f}}-{\rm{s}}{\rm{u}}{\rm{n}}}^{max}$.

**Figure 5:**
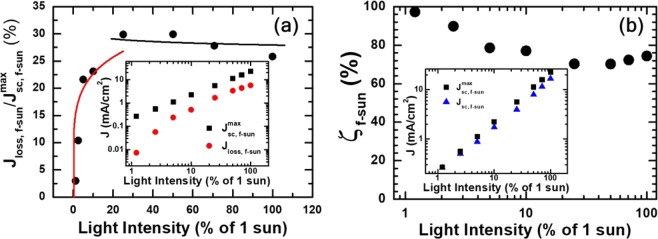
(**a**) Ratio of loss currents to maximum short-circuit currents with respect to light intensity. The maximum short-circuit currents were estimated by assuming *IQE* of 1 in the wavelength range from *λ*_min_ to *λ*_max_. Loss currents were defined as the difference between the maximum and measured short-circuit currents. Inset of (**a**) shows the maximum short-circuit and loss currents versus light intensity. Because of the power-law dependence of loss currents on intensity, given as *f*^*β*^ where *β* is an exponent that depends on recombination order, the variation of loss-current ratio with respect to intensity must be proportional to *f*^*β*−1^. Red and black lines are fitting curves corresponding to *β* values of 1.18 and 1.02, respectively. (**b**) *IQE* was determined as the ratio of measured *J*_sc,f-sun_ to the estimated maximum short-circuit current. Inset of (**b**) shows good agreement between the measured and estimated values of *J*_sc_, the latter of which was obtained under constant-*IQE* approximation.

By combining ${J}_{{\rm{s}}{\rm{c}},{\rm{f}}-{\rm{s}}{\rm{u}}{\rm{n}}}^{max}\propto f$ and ${J}_{{\rm{loss}},{\rm{f}}-{\rm{sun}}}\propto {f}^{\beta }$ with a recombination-order exponent *β*, we came to the conclusion that ${J}_{{\rm{l}}{\rm{o}}{\rm{s}}{\rm{s}},{\rm{f}}-{\rm{s}}{\rm{u}}{\rm{n}}}/{J}_{{\rm{s}}{\rm{c}},{\rm{f}}-{\rm{s}}{\rm{u}}{\rm{n}}}^{max}\propto \sim {f}^{\beta -1}$. Indeed, the intensity-dependent variation of ${J}_{{\rm{l}}{\rm{o}}{\rm{s}}{\rm{s}},{\rm{f}}-{\rm{s}}{\rm{u}}{\rm{n}}}/{J}_{{\rm{s}}{\rm{c}},{\rm{f}}-{\rm{s}}{\rm{u}}{\rm{n}}}^{max}$ was proportional to *f*^*β*−1^ as confirmed by the solid lines in Fig. 5(a). The red and black lines are fitting curves of ${J}_{{\rm{l}}{\rm{o}}{\rm{s}}{\rm{s}},{\rm{f}}-{\rm{s}}{\rm{u}}{\rm{n}}}/{J}_{{\rm{s}}{\rm{c}},{\rm{f}}-{\rm{s}}{\rm{u}}{\rm{n}}}^{max}$ to *f*^*β*−1^ with *β* values of 1.18 and 1.02 for low- and high-illumination operations, respectively. Typically, a recombination-order exponent *β* is related to an ideality factor *n* in diode-like devices as *β* = 2/*n*^36,50^. Interestingly, the calculated *n* value of 1.69 corresponding to low-illumination operations was close to those in Fig. 4(d). Similarly, we found that *n* value of 2.05 was consistent with the range of high-illumination *n* in Fig. 4(d).

Good agreement between *n* values determined from the fitting of intensity-dependent ${J}_{{\rm{l}}{\rm{o}}{\rm{s}}{\rm{s}},{\rm{f}}-{\rm{s}}{\rm{u}}{\rm{n}}}/{J}_{{\rm{s}}{\rm{c}},{\rm{f}}-{\rm{s}}{\rm{u}}{\rm{n}}}^{max}$ with those of *J*-*V* curves indicate that the constant-*IQE* approximation we used was appropriate for the estimation of short-circuit and/or loss currents. Validity of the constant-*IQE* approximation was further supported by good agreement between the measured and estimated values of *J*_sc_ shown in the inset of Fig. 5(b). Short-circuit currents estimated using Eq. (2) showed good quantitative agreement with the measured values. We can conversely estimate *ζ*_f-sun_, from the ratio of *J*_sc__,__f-sun_to ${J}_{{\rm{s}}{\rm{c}},{\rm{f}}-{\rm{s}}{\rm{u}}{\rm{n}}}^{max}$ (see [1] in Supplementary Information) provided that the constant-*IQE* approximation is valid. Constant *IQE* values estimated as ${\zeta }_{{\rm{f}}-{\rm{s}}{\rm{u}}{\rm{n}}}={J}_{{\rm{s}}{\rm{c}},{\rm{f}}-{\rm{s}}{\rm{u}}{\rm{n}}}/{J}_{{\rm{s}}{\rm{c}},{\rm{f}}-{\rm{s}}{\rm{u}}{\rm{n}}}^{max}$ are shown in Fig. 5(b). *ζ*_f-sun_ decreased monotonically in low-illumination range, from 97% at 0.01 sun to 77% at 0.1 sun but changed only slightly in high-illumination range and was about 74% at 1 sun^29^.

**Figure 6:**
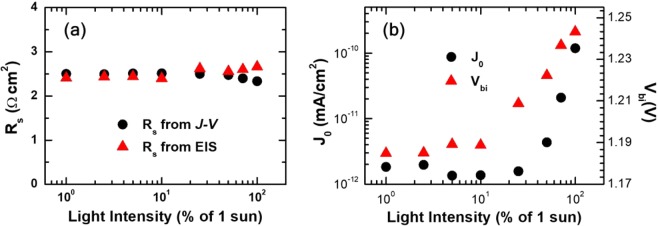
Variations of series resistance *R*_s_, reverse saturation current *J*_0_, and built-in potential *V*_bi_ with respect to light intensity. (**a**) Values of *R*_s_ determined from the fitting of *J*-*V* characteristics showed reasonably good agreement with those from the fitting of EIS spectra in Fig. 2. Both sets of *R*_s_ values showed no significant dependence on light intensity. (**b**) Values of *J*_0_ determined from the fitting of *J*-*V* characteristics to the modified Shockley-equation approximation, and those of *V*_bi_ that were estimated by using *J*_0_ and *n* in Fig. 4(d).

Other parameter values that we were able to determine from fitting the *J*-*V* characteristics to the modified Shockley-equation approximation were those of series resistance *R*_s_ and reverse saturation currents *J*_0_. As shown in Fig. 6(a), the value of *R*_s_ hardly changed with respect to light intensity. Lack of significant dependence on light intensity is an expected trait of parasitic series resistances. Moreover, another set of *R*_s_ values estimated by converting *R*_0_ values determined from the fitting of EIS spectra to an equivalent circuit (see Fig. 2) showed good agreement with those directly determined from the *J*-*V* fitting: ${R}_{{\rm{s}}}^{{\rm{E}}{\rm{I}}{\rm{S}}}={R}_{0}(V)\times S$ where *S* is the device area. We argue that good agreement between the values of *R*_s_ determined from the *J*-*V* fitting with those estimated from the EIS fitting is strong evidence supporting the validity of the modified Shockley-equation model that we used.

We present the variation of *J*_0_ with respect to light intensity in Fig. 6(b). Contrary to an almost constant *R*_s_, *J*_0_ showed an intensity-dependent exponential increase under high illumination. In combination with the variation of ideality factor *n* in Fig. 4(d), such increase of *J*_0_ resulted in noticeable increase of built-in potential *V*_bi_ for illumination above 0.25 sun as shown in Fig. 5(b). Specifically, built-in potential of devices showing rectifying behavior depends on *J*_0_ and *n*: *V*_bi_ = *A* − *nV*_th_(ln *J*_0_) where *V*_th_ is thermal voltage *k*_*B*_*T*/*q*^53–55^. Because constant *A* is typically much smaller than the second term regardless of junction types, values of built-in potential was estimated using the approximation of *V*_bi_ ≈ −*nV*_th_(ln *J*_0_)^54,55^. We argue that the distinct intensity dependence of *V*_bi_ is another evidence for the operation of two different mechanisms under low- and high-intensity illumination.

The intensity dependence of *V*_bi_ in Fig. 6(b) is seemingly different from that of *V*_oc_ in Fig. 1(d) because of additional contributions from *n* and *J*_sc_. In the modified Shockley-equation approximation, *V*_oc_ can be expressed in terms of *V*_bi_, *n*, and *J*_sc_ as following ${V}_{{\rm{oc}}}=n\,{V}_{{\rm{th}}}\,\mathrm{ln}\,({J}_{{\rm{sc}}})+{V}_{{\rm{bi}}}$^36^. For quantitative elucidation of the light-intensity dependence of *V*_bi_, we defined and examined the variation of $\Delta {V}_{{\rm{oc}},{\rm{1f}}}={V}_{{\rm{oc}},1-{\rm{sun}}}-{V}_{{\rm{oc}},{\rm{f}}-{\rm{sun}}}$ in terms of $\Delta {n}_{{\rm{1f}}}{={\rm{n}}}_{1-{\rm{sun}}}-{n}_{{\rm{f}}-{\rm{sun}}}$, $\Delta {V}_{{\rm{bi}},{\rm{1f}}}={V}_{{\rm{bi}},1-{\rm{sun}}}-{V}_{{\rm{bi}},{\rm{f}}-{\rm{sun}}}$, ${J}_{{\rm{sc}},1-{\rm{sun}}}/{J}_{{\rm{sc}},{\rm{f}}-{\rm{sun}}}$, and ${n}_{{\rm{f}}-{\rm{sun}}}$ (see [4] in Supplementary Information):

3 $$\begin{array}{rcl}\Delta {V}_{{\rm{oc}},{\rm{1f}}} & = & \Delta {n}_{{\rm{1f}}}\cdot {V}_{{\rm{th}}}\cdot \,\mathrm{ln}\,({J}_{{\rm{sc}},1-{\rm{sun}}})+\Delta {V}_{{\rm{bi}},{\rm{1f}}}+{n}_{{\rm{f}}-{\rm{sun}}}\,{V}_{{\rm{th}}}\,\mathrm{ln}(\frac{{J}_{{\rm{sc}},1-{\rm{sun}}}}{{J}_{{\rm{sc}},{\rm{f}}-{\rm{sun}}}})\\  & = & \Delta {V}_{{\rm{oc}}-1}^{{\rm{cal}}}+\Delta {V}_{{\rm{oc}}-2}^{{\rm{cal}}}+\Delta {V}_{{\rm{oc}}-3}^{{\rm{cal}}}\end{array}$$

**Figure 7:**
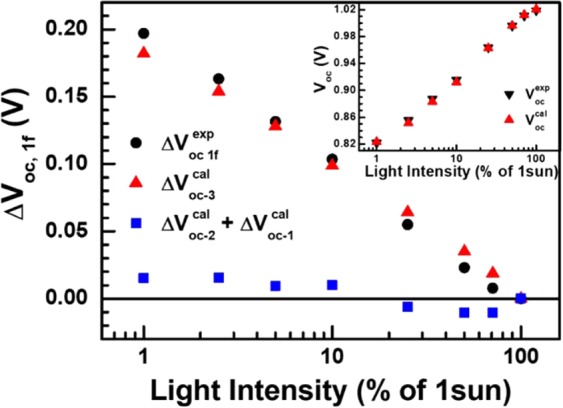
Variations of *V*_oc_ and Δ*V*_oc,1f_ that is defined as Δ*V*_oc,1f_ = Δ*V*_oc,1−sun_ − Δ*V*_oc,f-sun_ with respect to light intensity. Inset shows good agreement of *V*_oc_ calculated by using the modified Shockley-equation approximation to those determined from the measured *I-V* curves. Δ*V*_oc,1f_ consists of three terms, two of which represent contributions from respective variations of ideality-factor, Δ*n*_1f_ = n_1−sun_ − *n*_f−sun_, and built-in potential, Δ*V*_bi,1f_ = Δ*V*_bi,1−sun_ − *V*_bi,f-sun_. On the contrary, the third term depends explicitly on *n*_f-sun_ and *J*_sc,f-sun_. The sum of the first two terms is much smaller than the contribution of the third term in low-illumination range. Consequently, the third term accounts for most of the Δ*V*_oc,1f_ variation. However, there is a noticeable discrepancy between measured Δ*V*_oc,1f_ and the third term in high-illumination range.

Interestingly, the sum $\Delta {V}_{{\rm{oc}}-1}^{{\rm{cal}}}+\Delta {V}_{{\rm{oc}}-2}^{{\rm{cal}}}$ is much smaller than $\Delta {V}_{{\rm{oc}}-3}^{{\rm{cal}}}$ in low-illumination range as shown in Fig. 7. Therefore, mototonically decreasing $\Delta {V}_{{\rm{oc}}-3}^{{\rm{cal}}}$accounts for most of $\Delta {V}_{{\rm{oc}},{\rm{1f}}}$ variation in this range. However, $\Delta {V}_{{\rm{oc}}-3}^{{\rm{cal}}}$ shows a more subtle variation in high-illumination range because of noticeable increase in *n*_f-sun_ together with more prominant decrease in $\mathrm{ln}({J}_{{\rm{sc}},1-{\rm{sun}}}/{J}_{{\rm{sc}},{\rm{f}}-{\rm{sun}}})$ (see Fig. S3 in the Supplementary Information). Consequently, $\Delta {V}_{{\rm{oc}}-3}^{{\rm{cal}}}$ becomes comparable in magnitude to $\Delta {V}_{{\rm{oc}}-1}^{{\rm{cal}}}+\Delta {V}_{{\rm{oc}}-2}^{{\rm{cal}}}$ in high-illumination range. Moreover, the contribution of $\Delta {V}_{{\rm{oc}}-1}^{{\rm{cal}}}+\Delta {V}_{{\rm{oc}}-2}^{{\rm{cal}}}$ to Δ*V*_oc,1f_ switches from positive in low-illumination range to negative in high-illumination range to result in noticeable deviation of Δ*V*_oc,1f_ from $\Delta {V}_{{\rm{oc}}-3}^{{\rm{cal}}}$. We argue that negative contribution of $\Delta {V}_{{\rm{oc}}-1}^{{\rm{cal}}}+\Delta {V}_{{\rm{oc}}-2}^{{\rm{cal}}}$ to Δ*V*_oc,1f_ in addition to noticeable increase in *n*_f-sun_ resulted in distinct slope change in semi-log plot of V_oc_ versus light intensity in Fig. 1.

**Figure 8:**
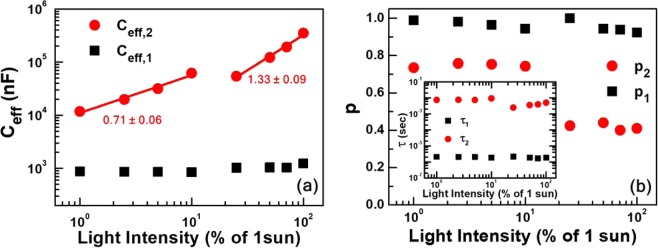
Light intensity-dependent variations of effective capacitance *C*_eff_ (**a**) and exponents *p* (**b**) of the two CPEs in the inset of Fig. 2. Values of *Q* and *p*, defining CPE impedance *Z*_CPE_ = *Q*(*jω*)^-*p*^, were determined from the fitting of impedance spectra to the equivalent circuit model. Effective capacitance *C*_eff,i_ was calculated by using the fitting-produced values of *Q*_*i*_, *p*_*i*_, and *R*_*i*_: *C*_*i*_ = [(*Q*_*i*_ *R*_*i*_)^1/*p*^_*i*_]/*R*_*i*_ for *i* = 1, 2. Each *R-C* pair represents a process with a characteristic time constant defined as *τ*_*i*_ = *R*_*i*_*C*_*i*_. Interestingly, *C*_eff,1_ and *p*_1_ varied only slightly with respect to intensity, and *p*_1_ remained larger than 0.93. On the contrary, *C*_eff,2_ and *p*_2_ showed significant difference between low- and high-illumination ranges.

Analysis of the equivalent-circuit parameters that we used to fit the EIS spectra in Fig. 2 provided additional evidence to support our hypothesis of intensity-dependent switching of internal processes, along with more detailed information on the processes itself. Figure 8(a,b) show equivalent capacitance *C*_eff_ and exponent *p* of the two CPEs, which we used to represent two processes with distinctly different characteristic times^37,40,41,56,57^. As the inset of Fig. 8(b) shows, the characteristic time of a fast process is about three orders of magnitude smaller than that of a slow process. The most prominent characteristics of the fast process are that both *C*_eff_ and *p* do not change significantly with respect to light intensity, and, moreover, *p* remains only slightly smaller than 1, In other words, the CPE representing a fast process appears similar to an ordinary junction capacitance in a diode-like device^40^. On the contrary, *C*_eff_ of the slow-process CPE shows a noticeable intensity-dependent increase. Specifically, a log-log plot of *C*_eff_ versus light intensity consists of two linear parts that correspond to low- and high-illumination ranges, respectively. The slope of the lower part is 0.71 ± 0.06 while that of the upper part is 1.33 ± 0.06. Another prominent feature is that the magnitude of slow-process *C*_eff_ is one or two orders of magnitude larger than that of the fast process. Such a large *C*_eff_ is likely to be related to excessive charge accumulation at interface^29,43,45,46^.

Similarly, *p* of the slow-process CPE showed an abrupt change from ~0.75 to ~0.42 between low- and high-illumination ranges. We note that large deviation of *p* from 1 is indicative of large distribution in time constants of relevant processes^41,56,57^, which, in turn, can originate from inhomogeneity of spatial and energetic features that are responsible for specific processes^56,57^. However, *p* of ~0.42 is unusually low, and typical time-constant distribution due to some physical inhomogeneity is not sufficient to account for such a low value of *p*. On the contrary, *p* of ~0.42 allows alternative interpretation of CPE impedance because of an appreciable real part^40^. That is, CPE impedance can be divided into real and imaginary parts, and we can interpret the real part as a frequency-dependent resistance. Characteristics of such CPE element is not purely capacitive, but more appropriately described as the sum of capacitive and frequency-dependent resistive contributions. We argue that the resistive part is relevant to recombination-loss currents. Therefore, the drop of *p* for light intensity larger than 0.25 sun, corresponding to the reduction of resistive contribution, is indicative of larger recombination-loss currents in high-illumination range.

**Figure 9:**
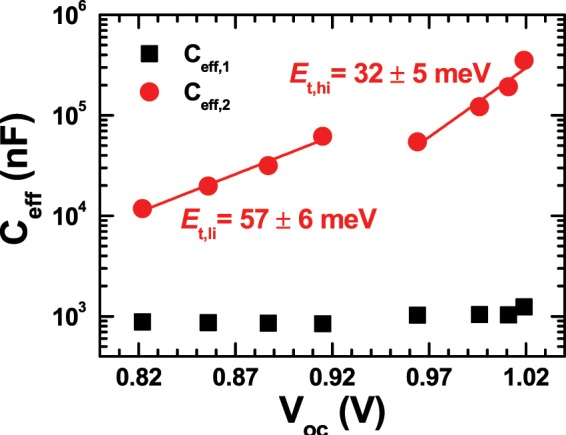
Effective capacitance *C*_eff_ versus open-circuit voltage *V*_oc_. Contrary to the effective capacitances of the first (fast-process) CPE, those of the second (slow-process) CPE shows appreciable increase with respect to open-circuit voltages. Slopes in a semi-log plot of *C*_eff_ versus *V*_oc_ correspond to tailing parameters *E*_t_ that characterize tail-like exponential distribution of defects.

Previously, we reported the effects of defects in PSC operation^37^. In particular, we attributed contributions of capacitive elements in EIS spectra to bulk and/or interface defects. We further expand the study on multiple types of defects by combining light intensity-dependent variations of *V*_oc_ and *C*_eff_. Semi-log plots of *C*_eff_ versus *V*_oc_ in Fig. 9 were constructed by combining *V*_oc_ in Fig. 1(d) and *C*_eff_ in Fig. 8(a). Linear increase in semi-log plots of *C*_eff_ versus *V*_oc_ can be attributed to defects with tail-like exponential distributions because capacitance due to such defects can be expressed in terms of a tailing parameter *E*_t_ characterizing exponential distributions as^43,58^:

4 $${C}_{i}={C}_{i,0}\exp (\frac{q{V}_{{\rm{o}}{\rm{c}}}}{{E}_{t,i}})\cdot $$

Hence, two linear segments in Fig. 9 was indicative of two different types of defects with tail-like exponential distributions. Tailing parameters corresponding to low- and high-illumination operations were 57 ± 6 and 32 ± 5 meV, respectively. In other words, the wide-distribution defects were main recombination centers in the PSC under low illumination, but the narrow-distribution defects took over charge recombination under high illumination.

Both the wide- and narrow-distribution defects had ionic origins, thus their contribution to the slow process was attributable to ionic motion^6,7,43–45^. However, their contributions to recombination loss were distinctly different. Arguably, the effect of wide-distribution defects as recombination centers did not overwhelm bimolecular recombination under low illumination, such that both bi- and mono-molecular recombination contributed to loss currents and resultant ideality factor of ~1.7^32,36^. On the contrary, the narrow-distribution defects became very effective as recombination centers under high illumination. Consequently, loss currents appeared to originate mostly from monomolecular recombination. There could be a third type of defect that made drift currents to be SCLC. From the exponent *η* of the power-law SCLC^49^, we were able to estimate another energy width that characterize the third energy distribution. If we assume a similar tail-like exponential distribution, the variation of *η* in Fig. 4 corresponds to that of the third tailing parameter ranging from 9.8 to 2.2 meV^49^.

## Conclusion

In this work, we showed that the *J*-*V* characteristics of a PSC under various illumination intensities ranging from 0.01 to 1 sun could be fitted to SCLC and modified Shockley-equation models to account for drift- and diffusion-dominant currents, respectively. Two main parameters that we determined from these fitting procedures were the power-law exponent *η* of a SCLC model and an ideality factor *n* in a modified Shockley equation. Both *η* and *n* showed distinct dependence on illumination intensity. Importance of non-ohmic drift currents was two-fold. First, SCLC currents were indicative of the contribution of defects in PSC operation. Second, it was required to analyze diffusion-dominant diode-like currents with discretion. Specifically, we isolated and fitted only diffusion-dominant diode-like currents to a modified Shockley-equation approximation. The validity of parameter values determined from the fitting procedure was crosschecked by comparing measured and estimated open-circuit voltages. Agreement of recombination-loss orders corresponding to fitting-produced *n* with those determined directly from light intensity-dependent variation of loss-current ratio was another source of validation. It is worth emphasizing that the approximation of constant *IQE* was used to estimate loss currents and open-circuit voltages. Successful use of a modified Shockley-equation model with a constant-*IQE* approximation led us to a new simple procedure to estimate *IQE* at each illumination intensity. To our knowledge, this simplified procedure for rough estimation of *IQE* values has not been reported previously.

Most importantly, we reported that light intensity-dependent variations of *η* and *n* resulted from the contribution of multiple types of defects in the PSC. It appeared that at least three types of defects with respectively discernible distributions contributed to distinct effects on the transport and recombination of charge carriers. The first type of defect, the effect of which was most prominent in a low bias-voltage range, contributed as charge traps to alter charge-carrier transport. We argue that this type of defects became progressively inactive as more charge carriers were generated with the increase of light intensity, and, eventually, drift currents became nearly ohmic under high illumination. On the other hand, the other two types of defects contributed mostly as recombination centers. Both defects had ionic origins and tail-like exponential distributions in common, but their contributions to recombination-loss currents showed discernably different dependence on light intensity. Consequently, we were able to determine respective tailing parameters that characterize two defect distributions. However, there was insufficient information to specify the three types of defects further, and identification of these defects remains a challenge.

## Methods

Planar MAPbI_3_ SCs with a structure of FTO/Cu-doped NiO_x_/MAPbI_3_/PCBM/LiF/Al were fabricated on a FTO-coated (7.33 Ω sq^−1^.) glass substrate. FTO-anode and LiF/Al-cathode patterns defined the rectangular solar-cell area of 0.4 × 0.6 cm^2^. FTO was patterned by using zinc powder and hydrochloric acid. On the contrary, LiF/Al was patterned by successive evaporation of LiF and Al through a shadow mask. Cu-doped NiO_x_ and PCBM were hole- and electron-extraction layers, respectively. A precursor solution of Cu-doped NiO_x_ was prepared by adding copper acetate monohydrate to a stock solution that was an equimolar mixture of Ni(CH_3_COO)_2_·4H_2_O and ethanolamine in 2-methoxyethanol. The molar ratio of Cu to Ni in the precursor solution was set at 10%. The precursor solution was spin coated onto a FTO-glass substrate, which was pretreated with UV-ozone for 30 min, at 4,000 rpm for 30 s. Annealing at 550 °C for 30 min in air transformed spin-coated precursor into a Cu-doped NiO_x_ layer, the thickness of which was determined as 17.06 ± 0.07 nm by using spectroscopic ellipsometry (see Fig. S4 in the Supplementary Information). The active layer of MAPbI_3_ was made by combining single-step precursor coating with subsequent toluene dripping. A precursor solution of MAPbI_3_ was prepared by dissolving the same amount of PbI_2_ and MAI in a mixture solvent of GBL and DMSO (7:3 v/v) under stirring at 60 °C for 12 h to result in the concentration of 1 mol L^−1^. The precursor solution of MAPbI_3_ was spin-coated onto the HEL/FTO substrate in two steps. The first spin-coating at 1,000 rpm for 10 s was followed by the second spin-coating at 5,000 rpm for 20 s, together with drop-casting of toluene. Subsequently, the substrate coated with MAPbI_3_ precursor was dried on a hot plate at 100 °C for 10 min to result in an active layer with the thickness of ~210 nm (see Fig. S5 in the Supplementary Information). All processing steps to make a MAPbI_3_ active layer were performed in a glove box. Next, a solution of Phenyl-C61-butyric acid methyl ester (PCBM) in chlorobenzene (20 mg ml^−1^) was spin-coated on the MAPbI_3_/HEL/FTO substrate at 1000 rpm for 30 s, and annealed at 110 °C for 10 min in a glove box. Finally, the fabricated SC was encapsulated by using glass cover with an ultraviolet (UV)-curable epoxy sealant (XNR5570-A1 NAGASE ChemTex). UV exposure time was ~2 min.

*J*-*V* characteristics under illumination were measured at a scan rate of 50 mV s^−1^ by using a Ivium CompactStat source-measuring unit. A PEC-L01 Peccell solar simulator operating at 100 mW cm^−2^ was used, together with a set of neutral filters, to simulate various illumination levels under AM1.5G condition. An aperture area of 0.15 cm^2^ was placed over the device during *J*-*V* measurements. EIS measurements were performed in open-circuit conditions by using the Ivium CompactStat system that was additionally equipped with a frequency analyzer module. We kept AC oscillating amplitude as low as 50 mV (rms) to maintain the linearity of the response. Prior to each *J*-*V* and EIS measurement, the device went through a pre-poling process at 0.8 V in dark for 100 s to restore the MAPbI_3_ active layer to the same condition. Moreover, *J*-*V* characteristics were measured only under forward bias-voltage sweep.

## Supplementary information

Supplementary Information
