# Author Correction: Light Intensity-dependent Variation in Defect Contributions to Charge Transport and Recombination in a Planar MAPbI_3_ Perovskite Solar Cell

**DOI:** 10.1038/s41598-020-59787-6

**Published:** 2020-03-04

**Authors:** Shinyoung Ryu, Duc Cuong Nguyen, Na Young Ha, Hui Joon Park, Y. H. Ahn, Ji-Yong Park, Soonil Lee

**Affiliations:** 1 Department of Energy Systems Research, Ajou University, Suwon, 16499 Korea; 2 Faculty of Engineering Physics and Nanotechnology, VNU University of Engineering and Technology, Vietnam National University, Hanoi, Vietnam; 3 Department of Physics, Ajou University, Suwon, 16499 Korea; 4 Department of Electrical and Computer Engineering, Ajou University, Suwon, 16499 Korea

Correction to: *Scientific Reports* 10.1038/s41598-019-56338-6, published online 27 December 2019

A glitch in the authors’ reference-management software led to extensive errors in the reference list and in-text citations. These errors have been corrected in the PDF and HTML versions of the Article.

