# Supplementary material for: Light Intensity-dependent Variation in Defect Contributions to Charge Transport and Recombination in a Planar MAPbI3 Perovskite Solar Cell
Source: Sci Rep. 2019 Dec 27;9:19846. doi: 10.1038/s41598-019-56338-6 (PMC6934867; doi:10.1038/s41598-019-56338-6)
Supplement: Supplementary file 1 — Supplementary Information [file 41598_2019_56338_MOESM1_ESM.doc]

**Supplementary Information**

**Light Intensity-dependent Variation in Defect Contributions to Charge Transport and Recombination in a Planar MAPbI3 Perovskite Solar Cell**

**Shinyoung Ryu^1, †^, Duc Cuong Nguyen^1,2,†^, Na Young Ha^1,3^, Hui Joon Park^1,4^, Y. H. Ahn^1,3^, Ji-Yong Park^1,3^, and Soonil Lee^1,3,*^**

1 Department of Energy Systems Research, Ajou University, Suwon 16499, Korea

2 Present Address: Faculty of Engineering Physics and Nanotechnology, VNU University of Engineering and Technology, Vietnam National University, Hanoi, Vietnam

3 Department of Physics, Ajou University, Suwon 16499, Korea

4 Department of Electrical and Computer Engineering, Ajou University, Suwon 16499, Korea

† Shinyoung Ryu, and Duc Cuong Nguyen contributed equally to this work.

* Address correspondence to Soonil Lee, soonil@ajou.ac.kr

**[1] Recombination-loss current and ratio**

Short-circuitcurrents can be estimated as follows:^1–3^

$J_{sc}=\int\frac{\lambda}{1240}\Phi_{sol}(\lambda)\cdot LHE(\lambda)\cdot IQE(\lambda)d\lambda=\int\frac{\lambda}{1240}\Phi_{sol}(\lambda)\cdot EQE(\lambda)d\lambda\text{ (where }\lambda\text{ is in nm)}$.

From $\Phi_{sol,f\text{-sun}}(\lambda)=\text{ }f\cdot\Phi_{sol,\text{1-sun}}(\lambda)$, we get

$J_{sc,\text{f-sun}}=f\cdot\int\frac{\lambda}{1240}\Phi_{1\text{-sun}}(\lambda)\cdot EQE_{\text{f-sun}}(\lambda)d\lambda$.

If we assume

$IQE_{\text{f-sun}}(\lambda)=\left[\begin{matrix} \zeta_{\text{f-sun}}\text{ (constant) for }\lambda_{\min}\leq\lambda\leq\lambda_{\max} \\ 0\text{ for }\lambda_{\min}\geq\lambda\text{ or }\lambda_{\max}\leq\lambda\end{matrix}\right.$,

then $J_{sc,\text{f-sun}}=f\cdot\zeta_{\text{f-sun}}\cdot\int_{\lambda_{\min}}^{\lambda_{\max}} \frac{\lambda}{1240}\Phi_{1\text{-sun}}(\lambda)LHE(\lambda)d\lambda$,

Accordingly,

$$\begin{matrix} J_{sc,1\text{-sun}}^{\max}=\int_{\lambda_{\min}}^{\lambda_{\max}} \frac{\lambda}{1240}\Phi_{1\text{-sun}}(\lambda)LHE(\lambda)d\lambda\text{ (when }\zeta_{\text{1-sun}}=1,\text{ }f=1) \\ J_{sc,\text{f-sun}}^{\max}=f\cdot\int_{\lambda_{\min}}^{\lambda_{\max}} \frac{\lambda}{1240}\Phi_{1\text{-sun}}(\lambda)LHE(\lambda)d\lambda=f\cdot J_{sc,1\text{-sun}}^{\max}\text{ (when }\zeta_{\text{f-sun}}=1,\text{ }f<1) \\ J_{sc,\text{f-sun}}=f\cdot\zeta_{\text{f-sun}}\cdot\int_{\lambda_{\min}}^{\lambda_{\max}} \frac{\lambda}{1240}\Phi_{1\text{-sun}}(\lambda)LHE(\lambda)d\lambda\\ =f\cdot\zeta_{\text{f-sun}}\cdot J_{sc,1\text{-sun}}^{\max}=\zeta_{\text{f-sun}}\cdot J_{sc,\text{f-sun}}^{\max}\text{ (when }\zeta_{\text{f-sun}}<1,\text{ }f<1) \\ J_{sc,\text{1-sun}}=\zeta_{\text{1-sun}}\cdot\int_{\lambda_{\min}}^{\lambda_{\max}} \frac{\lambda}{1240}\Phi_{1\text{-sun}}(\lambda)LHE(\lambda)d\lambda=\zeta_{\text{1-sun}}\cdot J_{sc,1\text{-sun}}^{\max}\text{ (when }\zeta_{\text{f-sun}}<1,\text{ }f=) \end{matrix}$$

Recombination loss currents ($J_{\text{loss,f-sun}}$) and ratios ($1-\zeta_{\text{f-sun}}$) at f-sun are given as following:

$$\begin{matrix} J_{\text{loss,f-sun}}=J_{sc,\text{f-sun}}^{\max}-J_{sc,\text{f-sun}} =(f-f\cdot\zeta_{\text{f-sun}})\cdot\int_{\lambda_{\min}}^{\lambda_{\max}} \frac{\lambda}{1240}\Phi_{1\text{-sun}}(\lambda)LHE(\lambda)d\lambda\\ =(1-\zeta_{\text{f-sun}})\cdot f\cdot\int_{\lambda_{\min}}^{\lambda_{\max}} \frac{\lambda}{1240}\Phi_{1\text{-sun}}(\lambda)LHE(\lambda)d\lambda=(1-\zeta_{\text{f-sun}})\cdot J_{sc,\text{f-sun}}^{\max} \\ J_{\text{loss,1-sun}}=J_{sc,1\text{-sun}}^{\max}-J_{sc,1\text{-sun}} =(1-\zeta_{\text{1-sun}})\cdot J_{sc,\text{1-sun}}^{\max} \end{matrix}$$

Inversely, recombination loss ratios can be given in terms of recombination loss currents:

$$\begin{matrix} \therefore\text{ }1-\zeta_{\text{1-sun}} =\frac{J_{\text{loss,}1\text{-sun}}}{J_{sc,1\text{-sun}}^{\max}}=\frac{J_{sc,1\text{-sun}}^{\max}-J_{sc,1\text{-sun}}}{J_{sc,1\text{-sun}}^{\max}}=1-\frac{J_{sc,1\text{-sun}}}{J_{sc,1\text{-sun}}^{\max}} \\ 1-\zeta_{\text{f-sun}} =\frac{J_{\text{loss,f-sun}}}{J_{sc,\text{f-sun}}^{\max}}=\frac{J_{sc,\text{f-sun}}^{\max}-J_{sc,\text{f-sun}}}{J_{sc,\text{f-sun}}^{\max}}=1-\frac{J_{sc,\text{f-sun}}}{J_{sc,\text{f-sun}}^{\max}}=1-\frac{J_{sc,\text{f-sun}}}{f\cdot J_{sc,\text{1-sun}}^{\max}} \end{matrix}$$

We approximated IQE spectra as a rectangular shape with a constant IQE value because EQE spectra of PSCs show roughly trapezoidal shape with rather steep drops at short- and long-wavelength ranges, and EQE and LHE spectra show only slight variations in the wavelength range responsible for most of photocurrents.^4, 5^

**[2] Effective ideality factor for combinations of mono- and bimolecular recombination^6^**

$$\begin{matrix} J_{\text{sum}}(V)=J_{\text{diff}}(V)+J_{\text{rec}}(V) \\ =J_{\text{diff}}(V)+J_{\text{rec}}^{\text{bi}}(V)+J_{\text{rec}}^{\text{mono}}(V) \\ =J_{\text{diff,0}}\left[\exp\left(\frac{qV}{k_{B}T}\right)-1\right]+J_{\text{rec,0}}^{\text{bi}}\left[\exp\left(\frac{qV}{k_{B}T}\right)-1\right]+J_{\text{rec,0}}^{\text{mono}}\left[\exp\left(\frac{qV}{2 k_{B}T}\right)-1\right] \\ =J_{\text{dibi,0}}\left[\exp\left(\frac{qV}{k_{B}T}\right)-1\right]+J_{\text{rec,0}}^{\text{mono}}\left[\exp\left(\frac{qV}{2 k_{B}T}\right)-1\right] \\ =J_{\text{dibi}}(V)+J_{\text{rec}}^{\text{mono}}(V) \\ \approx\text{ }J_{0}\left[\exp\left(\frac{qV}{n k_{B}T}\right)-1\right] \end{matrix}$$

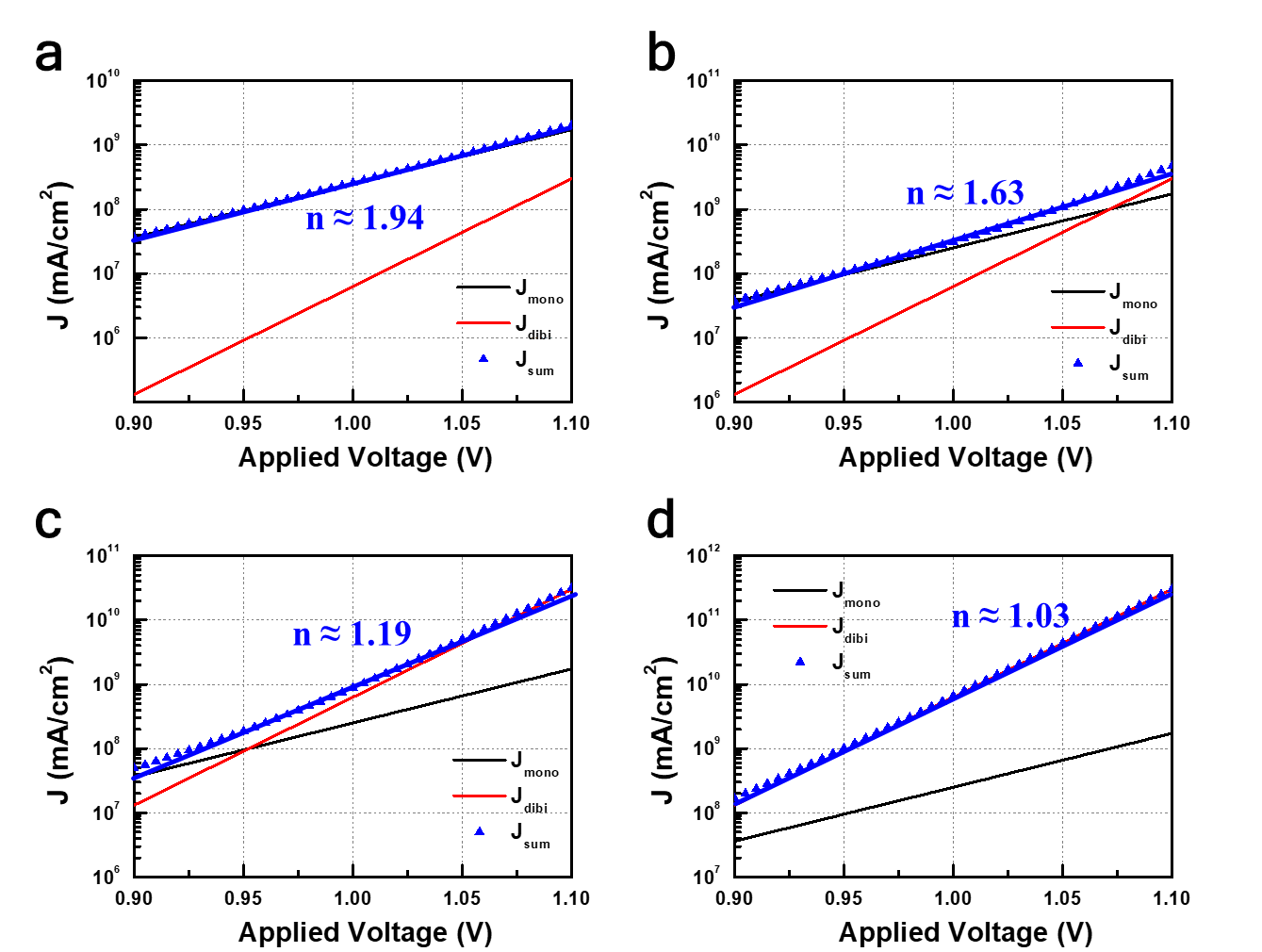


**Figure S1.** Addition of monomolecular-recombination currents to conventional exponential currents. Such combination can be approximated using a modified Shockley equation with an ideality factor *n*, the values of which lies between 1 and 2.

**[3] Fittings of *J-V* curves to SCLC and modified Shockley-equation models**


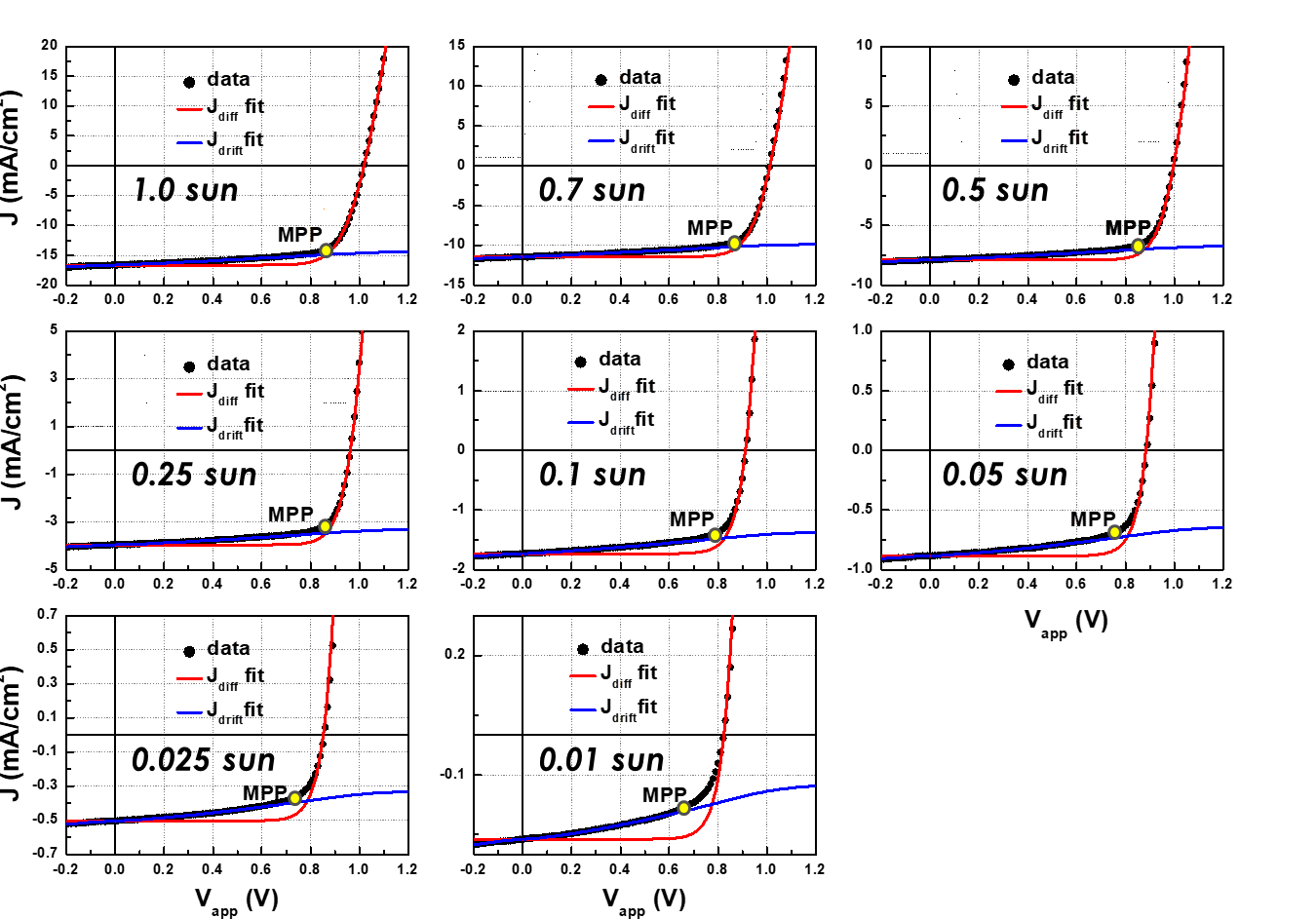


**Figure S2.** Separate fittings of low- and high-bias sections of *J-V* curves corresponding to different light intensities to a SCLC model and a modified Shockley equation, respectively. Space-charge-limited drift currents dominated *J-V* characteristics in low-bias ranges below the maximum power point (MPP) at each illumination. However, exponential Shockley-like *J-V* characteristics became dominant in high-bias ranges above the MPP.

**[4] Variations of *V*_oc_ and parameters in the modified Shockley-equation approximation^6^**

From

$$\begin{matrix} J_{\text{sc}}=\text{ }J_{0}\left[\exp\left(V_{\text{oc}}/n V_{\text{th}}\right)-1\right]\approx J_{0} \exp\left(V_{\text{oc}}/n V_{\text{th}}\right) \\ \ln(J_{\text{sc}})=\ln(J_{0})+V_{\text{oc}}/n V_{\text{th}} \\ V_{\text{oc}}=n V_{\text{th}} \ln(J_{\text{sc}})-n V_{\text{th}} \ln(J_{0})=n V_{\text{th}}\ln(J_{\text{sc}})+V_{\text{bi}} \end{matrix}$$

We have

$$\begin{matrix} V_{\text{oc, 1-sun}}= n_{\text{1-sun}} V_{\text{th}}\ln(J_{\text{sc, 1-sun}})+V_{\text{bi, 1-sun}} \\ V_{\text{oc, f-sun}}=n_{\text{f-sun}} V_{\text{th}}\ln(J_{\text{sc, f-sun}})+V_{\text{bi, f-sun}} \\ =n_{\text{f-sun}} V_{\text{th}}\ln(J_{\text{sc, f-sun}})+n_{\text{f-sun}} V_{\text{th}}\ln(J_{\text{sc, 1-sun}})-n_{\text{f-sun}} V_{\text{th}}\ln(J_{\text{sc, 1-sun}})+V_{\text{bi, f-sun}} \\ =n_{\text{f-sun}} V_{\text{th}}\ln(J_{\text{sc, 1-sun}})-n_{\text{f-sun}} V_{\text{th}}\ln\left(\frac{J_{\text{sc, 1-sun}}}{J_{\text{sc, f-sun}}}\right)+V_{\text{bi, f-sun}} \end{matrix}$$

Therefore,

$$V_{\text{oc, 1-sun}}-V_{\text{oc, f-sun}}=(n_{\text{1-sun}} -n_{\text{f-sun}}) V_{\text{th}}\ln(J_{\text{sc, 1-sun}})+(V_{\text{bi, 1-sun}}-V_{\text{bi, f-sun}})+n_{\text{f-sun}} V_{\text{th}}\ln\left(\frac{J_{\text{sc, 1-sun}}}{J_{\text{sc, f-sun}}}\right)$$

If we define ,

$\Delta V_{\text{oc, 1f}}=\text{ }V_{\text{oc, 1-sun}}-V_{\text{oc, f-sun}}\text{, }\Delta n_{\text{1f}}=\text{ }n_{\text{1-sun}} -n_{\text{f-sun}}\text{, and }\Delta V_{\text{bi, 1f}}=V_{\text{bi, 1-sun}}-V_{\text{bi, f-sun}}$,

Then

$$\Delta V_{\text{oc, 1f}}=\Delta n_{\text{1f}}\cdot V_{\text{th}}\cdot\ln(J_{\text{sc, 1-sun}})+\Delta V_{\text{bi, 1f}}+n_{\text{f-sun}} V_{\text{th}}\ln\left(\frac{J_{\text{sc, 1-sun}}}{J_{\text{sc, f-sun}}}\right)$$

Figure S3. Contributions of n and ln (J_SC, 1-sun_ / J_SC, f-sun_) variations to the illumination-intensity dependence of ΔV_OC3_ / V_th_. ln (J_SC, 1-sun_ / J_SC, f-sun_) decreases monotonically and qualitatively accounts for ΔV_OC3_ reduction with respect to illumination intensity. However, increase of n in high-intensity range has to be taken into account for quantitative assessment of ΔV_OC3_ variation.

**[5] Summary of perovskite solar cell operation under various illumination intensity**

**Table S1**. Illumination intensity dependence of operation parameters of the perovskite solar cell.

| Light Intensity  (mW cm^-2^) | V_oc_  (V) | J_sc_  (mA cm^-2^) | FF  (%) | PCE  (%) |
| --- | --- | --- | --- | --- |
| 100 | 1.019 | 16.7 | 71.5 | 12.17 |
| 70 | 1.011 | 11.5 | 72.3 | 11.87 |
| 50 | 0.996 | 7.90 | 72.3 | 11.35 |
| 25 | 0.964 | 3.96 | 71.8 | 10.92 |
| 10 | 0.915 | 1.73 | 69.5 | 11.00 |
| 5 | 0.887 | 8.82 × 10^-1^ | 67 | 10.48 |
| 2.5 | 0.856 | 5.04 × 10^-1^ | 62.8 | 10.84 |
| 1 | 0.822 | 2.62 × 10^-1^ | 56.1 | 12.08 |

**[6] AFM image and a Tauc plot of a Cu10:NiO_x_ layer**

| **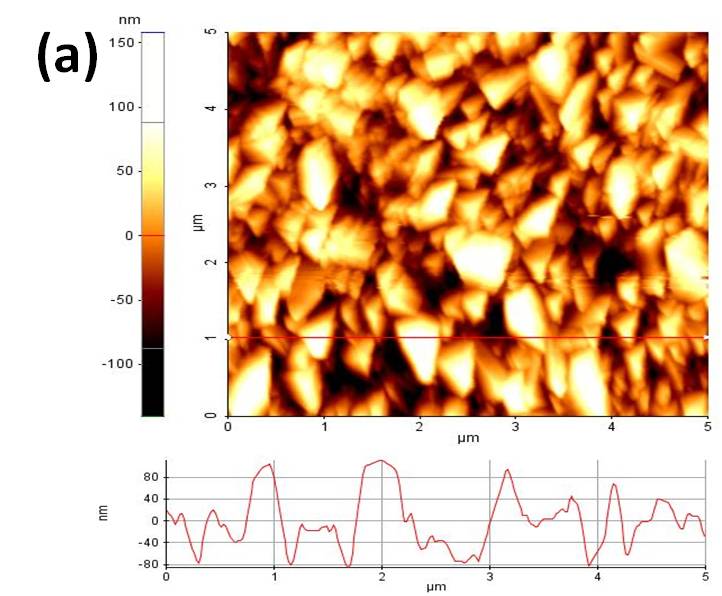** | **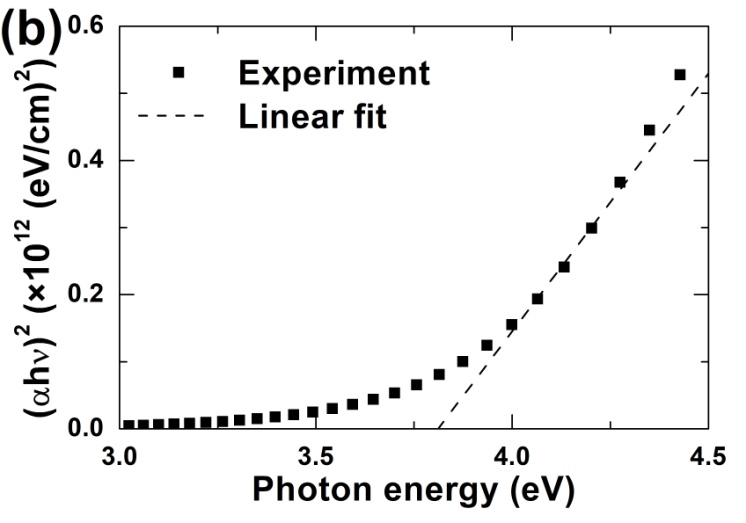** |
| --- | --- |

**Figure S4.** (a) Atomic Force Microscopy (AFM) image of a 10% Cu-doped NiOx (Cu10:NiOx) layer deposited on a FTO-coated glass substrate. Root-mean-square (RMS) roughness of a Cu10:NiO_x_ layer was estimated to be 50.1 nm. (b) Tauc plot of a Cu10:NiO_x_ layer. Absorption coefficients $\alpha(\lambda)$ were estimated by using extinction coefficients $k(\lambda)$ that were determined by analysing spectroscopic ellipsometry spectra: $\alpha(\lambda)=4\pi k/\lambda$.

**[7] XRD patterns and a SEM image of a CH_3_NH_3_PbI_3_ layer**

| 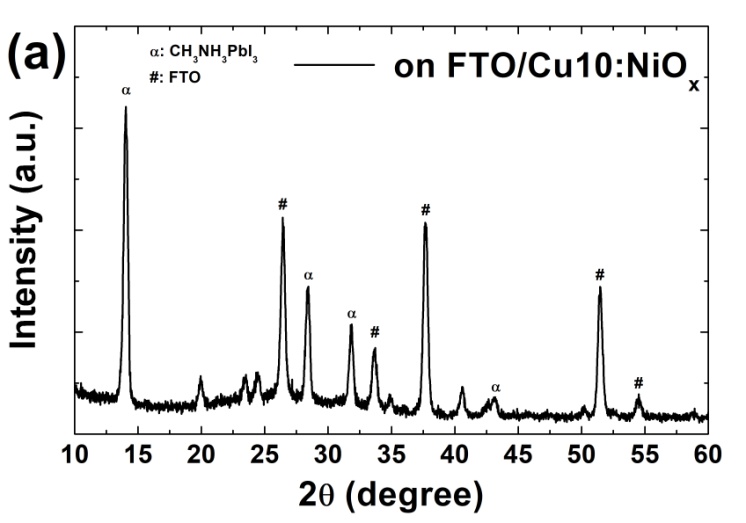 | 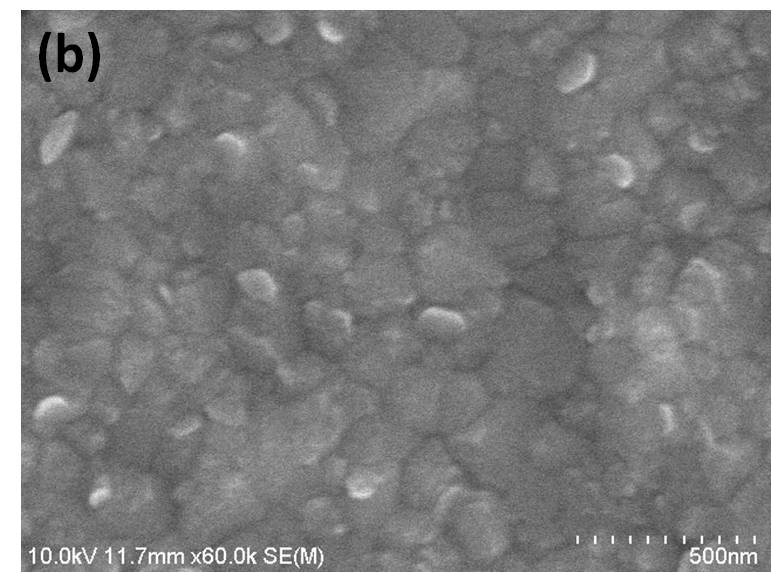 |
| --- | --- |

**Figure S5.** (a) X-ray diffraction (XRD) pattern of a CH_3_NH_3_PbI_3_ layer formed onto a Cu10:NiO_x_ layer deposited on a FTO-coated glass substrate. α denotes peaks corresponding to the tetragonal perovskite phase: (110) at 14.06°, (220) at 28.38°, (310) at 31.78°, and (330) at 43.18°. # denotes diffraction peaks due to a FTO layer. (b) Top-view scanning electron microscopy (SEM) image of a CH_3_NH_3_PbI_3_ layer on FTO/Cu10:NiO_x_.

**References**

1. Joe, S.-Y. *et al.* Universal efficiency improvement in organic solar cells based on a poly(3-hexylthiophene) donor and an indene-C60 bisadduct acceptor with additional donor nanowires. *ChemPhysChem* **16**, 1217–1222 (2015).

2. Joe, S.-Y. *et al.* Contributions of poly(3-hexylthiophene) nanowires to alteration of vertical inhomogeneity of bulk-heterojunction active layers and improvements of light-harvesting and power-conversion efficiency of organic solar cells. *Org. Electron. physics, Mater. Appl.* **42**, 372–378 (2017).

3. Nguyen, D. C. Effects of device structure and operating conditions on the performance of organometal trihalide perovskite solar cells: Device characterization and impedance spectroscopy study (Ajou University) (2017).

4. Xie, L., Hwang, H., Kim, M. & Kim, K. Ternary solvent for CH_3_NH_3_PbI_3_ perovskite films with uniform domain size. *Phys. Chem. Chem. Phys*. **19**, 1143-1150 (2017).

5. Barone, C. *et al.* Unravelling the low-temperature metastable state in perovskite solar cells by noise spectroscopy. *Sci. Rep.* **6**, 34675 (2016).

6. Jenny Nelson. The Physics of Solar Cells. 148–164 (Imperial College Press, 2003).
